# Triple combination MPT vaginal microbicide using curcumin and efavirenz loaded lactoferrin nanoparticles

**DOI:** 10.1038/srep25479

**Published:** 2016-05-06

**Authors:** Yeruva Samrajya Lakshmi, Prashant Kumar, Golla Kishore, C Bhaskar, Anand K Kondapi

**Affiliations:** 1 Department of Biotechnology and Bioinformatics, School of Life Sciences, University of Hyderabad, Hyderabad-500046, India

## Abstract

We report that a combination of anti-HIV-1 drug efavirenz (EFV), anti-microbial-spermicidal curcumin (Cur) and lactoferrin nanoparticles (ECNPs) act as MPT formulation. These nanoparticles are of well dispersed spherical shape with 40–70 nm size, with encapsulation efficiency of 63 ± 1.9% of Cur & 61.5% ± 1.6 of EFV, significantly higher than that of single drug nanoparticles (Cur, 59 ± 1.34%; EFV: 58.4 ± 1.79). ECNPs were found to be sensitive at pH 5 and 6 and have not effected viability of vaginal micro-flora, *Lactobacillus*. Studies in rats showed that ECNPs delivers 88–124% more drugs in vaginal lavage as compared to its soluble form, either as single or combination of EFV and Cur. The ECNPs also shows 1.39–4.73 fold lower concentration of absorption in vaginal tissue and plasma compared to soluble EFV + Cur. Furthermore, ECNPs show significant reduction in inflammatory responses by 1.6–3.0 fold in terms of IL-6 and TNF-α in vaginal tissue and plasma compared to soluble EFV + Cur. ECNPs showed improved pharmacokinetics profiles in vaginal lavage with more than 50% of enhancement in AUC, AUMC, C_max_ and t_1/2_ suggesting longer exposure of Cur and EFV in vaginal lavage compared to soluble EFV + Cur. Histopathological analysis of vaginal tissue shows remarkably lower toxicity of ECNPs compared to soluble EFV + Cur. In conclusion, ECNPs are significantly safe and exhibit higher bioavailability thus constitute an effective MPT against HIV.

Across the globe, a major number of women, especially in developing countries, needs protection from various sexual transmitted infections (STIs) like HIV/AIDS as well as sexual and reproductive health (SRH) risk that includes unintended pregnancy. Further due to STIs, the women in low and middle income countries like sub-Saharan region experience about 41% of unintended pregnancies that leads to approximately 70,000 deaths^1^,^2^. Hence, there is an urgent need to develop technologies which can target these conditions at a time. The Multiple prevention technologies (MPT) are currently the most promising and intricate class of product under development which can simultaneously prevent transmission from the STIs like HIV/AIDS and unwanted pregnancy^3^. Currently three types of MPT are available; which include male and female condoms, female Diaphragms and microbicide (chemical barrier)^4^. Our current study focus on the vaginal microbicide based MPT.

Several research groups have already reported various combinations of microbicides^5^,^6^. Some of the formulations have undergone different stages of clinical trials, and a large numbers of formulations have failed due to safety, toxicity and efficacy issues^7^,^8^,^9^. In addition to this other microbicide such as PRO 2000, Carraguard (Phase III) and BufferGel were also not found to be successful at different phases of clinical trials^10^,^11^. The major requirement of a microbicide is, to release optimum concentrations of active drug in the vagina along with higher bioavailability for longer period without causing discomfort and adverse effects to the biological barriers. Polyanions and surfactants based vaginal microbicides against HIV were also not successful; while nonoxynol-9 has increased the risk of HIV acquirement^12^,^13^. In contrast, Anti-retroviral (ARV) drugs based microbicides have shown improved neutralization and thus are clinically more significant^14^. The main property of microbicide either as semi-solid gels, vaginal films, tablets, or ring is to provide a sustained release of drug over a longertime^15^. Further, Tenofovir (TDF) based intravaginal rings (IVR) provide a controlled release of TDF in to the vagina at a rate of 76.4 ± 54.8 μgg^−1^ and remained constant for seven days after the removal of IVR^15^. Duration of drug release is 8 hr for vaginal tablets^16^, 6 hr for vaginal gels^17^ and 72 hr for vaginal rings^18^.

Curcumin, an active principle of unique herbal compound turmeric has been used as the main spice ingredient in most parts of Asian subcontinent since centuries. It exhibits pleiotropic effect like anti-HIV^19^, anti-inflammatory^20^, anti-oxidant^21^, vaginal contraceptive^22^ and many more. Curcumin shows concentration-dependent inhibition of sperm motility and complete block at concentration of ≥250 μM^23^. But its hydrophobic nature and low bioavailability apparently put limitations on its use in conventional therapeutic application^24^. Many approaches were attempted to overcome these limitations; and nanoparticle mediated drug formulation is one of approaches investigated. Nanoparticles (NPs) size ranges from 1–100 nm, which themselves act as a whole unit that is capable of transport as well as release of drug^25^. NPs also provide several additional benefits, e.g. protection of the drugs against degradation, facilitates targeted action, and delivery of varieties of biological bits, like peptide, protein and nucleotides. They have the ability to overcome many classical drug delivery challenges such as controlled drug delivery, greater cellular acceptance of poorly permeable drugs, problems of physicochemical stability and reduction of immunogenic response. The therapeutic efficacy and the degree of safety of drugs can be considerably improved by targeted delivery using nanocarriers^26^,^27^. Many protein based nanoparticles have been already developed for therapeutic uses to treat cancer, AIDS and Parkinson’s^28^,^29^.

The main benefit of protein nanoparticles is that, these can be prepare in relatively mild condition without use of any toxic chemicals^29^,^30^,^31^. Protein could be an ideal vehicle for drug transportation in nano-form because its amphiphilic nature helps its cooperation with drugs as well as solvent^32^. Curcumin loaded-apotransferrin nanoparticles are shown to inhibit HIV-1 through inhibition of virus infection and down-regulation of host inflammatory responses^33^.

Natural protein such as lactoferrin; is water soluble, metabolizable, biodegradable; Surface modification of such natural protein could be done very easily to facilitate required interaction of drugs and ligands^34^. Lactoferrin protein has several pleiotropic functions like immune modulation, anti-viral, and anti-cancer, and thus can act as first line of defense to inhibit inflammation and infection^35^,^36^,^37^,^38^,^39^.

The objective of our study is to develop a triple-combination topical formulation that can simultaneously act on HIV, HIV-mediated inflammation, other viral and bacterial infections with contraceptive action, based on the principle of multipurpose prevention technologies (MPT). This is a triple combination of broad spectrum lactoferrin (as vehicle) and curcumin as preventive and protective agent and EFV as therapeutic agent against HIV. The principle steps in realizing the objectives of this studies are (1) preparation and characterization of lactoferrin nanoparticles loaded with curcumin and EFV; (2) Studying bioavailability and pharmacokinetic profile of curcumin and EFV in vaginal lavage upon topical application of nanoformulation, and (3) Evaluation of safety of nanoformulation in terms of inflammation.

## Results

### Preparation and characterization of efavirenz and curcumin loaded lactoferrin nanoparticle

**Figure 1:**
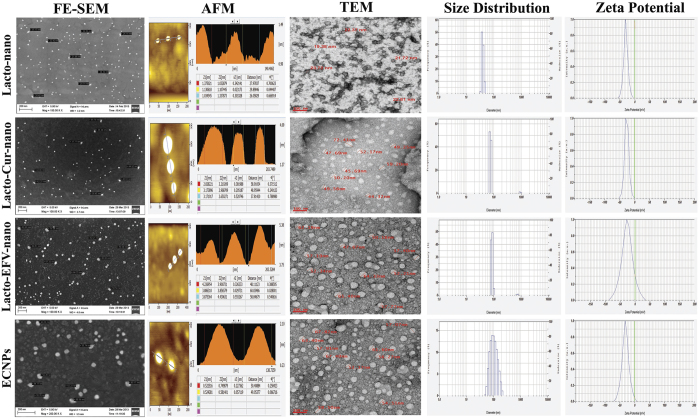
Microscopic and DLS analysis of nanoparticle. Left to right panel indicates the FE-SEM, AFM, TEM, size distribution and zeta potential. Top to bottom; Blank lactoferrin nanoparticle (Lacto-nano), Curcumin loaded lactoferrin nanoparticle (Lacto-Cur-nano or CNP), Efavirenz loaded lactoferrin nanoparticles (Lacto-EFV-nano or ENP), Cur+EFV loaded Lactoferrin nanoparticles (ECNPs).

Curcumin loaded Lactoferrin NPs (Lacto-Cur-nano), Efavirenz loaded Lactoferrin NPs (Lacto-EFV-nano) and Efavirenz plus curcumin loaded lactoferrin nanoparticles (ECNPs) were prepared using sol-oil chemistry as described in materials and methods section. The nanoparticles prepared were characterized through FE-SEM (Field Emission Scanning Electron Microscope), AFM (Atomic-force microscopy), TEM (Transmission electron microscopy) and DLS (Dynamic light scattering). Results presented in Fig. 1 shows that nanoparticles were uniformly dispersed spherical particles with size in the range of 40–70 nm. AFM images provide a three-dimensional surface profile reveal a particular type of projection which may help in binding with the receptor. DLS analysis of blank or drug/s loaded NPs has showed the hydrodynamic size in a range of 40 nm and 91–125 nm respectively. Increased apparent size in DLS is due to the surface water shell that contribute in DLS measurements. The zeta potential of freshly prepared blank or drug/s loaded NP were found to be in a range of −21 to −25 mv respectively (Supplementary Table 1) indicating their stability.

### FT-IR spectral analysis

**Figure 2:**
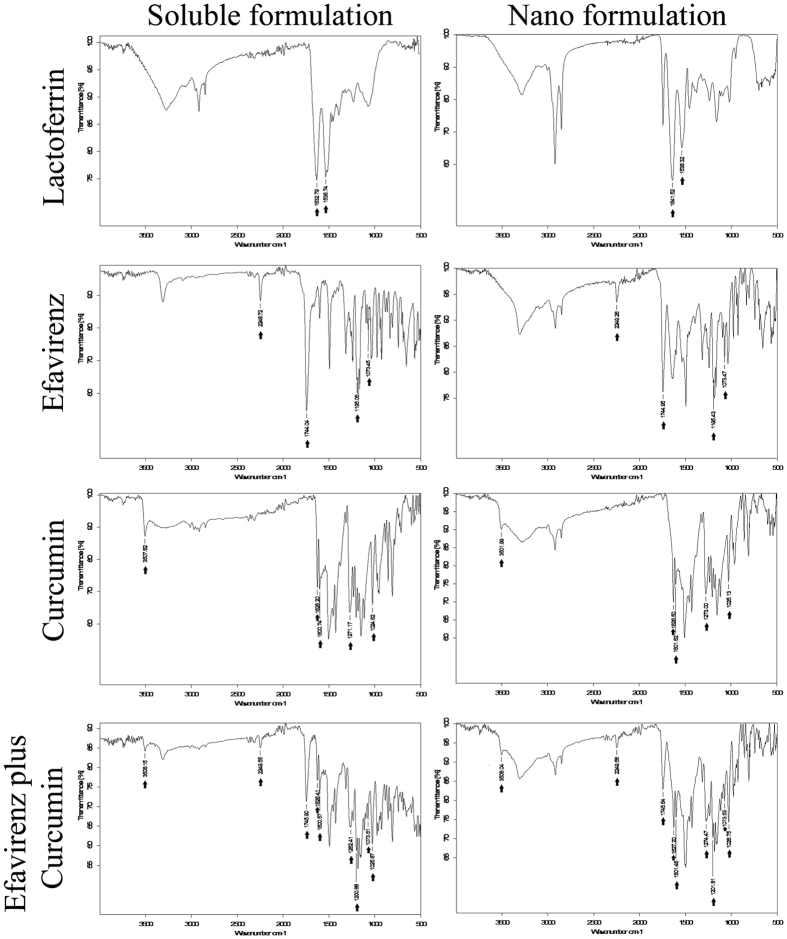
Fourier transform infrared spectroscopy (FTIR) spectral analysis of nanoparticle. Left and right panel indicate the spectra of soluble and nanoformulation respectively. Top to bottom: - Lactoferrin, Efavirenz, curcumin and combination of efavirenz and curcumin. All samples were lyophilized prior to scanning and data were collected using OPUS software.

FT-IR spectral data showed the stability of lactoferrin, efavirenz, curcumin and efavirenz plus curcumin combination which remained conserved in their nanoformulation (Fig. 2). All relevant FT-IR peaks related to the soluble and nanoformulation were indicated with black arrows and found to be almost identical with minor deviations in their intensities (Supplementary Table 2).

### Assessment of loading efficiency

Loading efficiency of Lacto-Cur-nano, Lacto-EFV-nano and ECNPs were assessed. ECNPs were prepared at four different concentrations of efavirenz by keeping the concentration of lactoferrin and curcumin constant (Table 1). Maximum loading was observed in formulation ratio II for ECNPs (63% ± 1.9 of Cur. and 61.5 ± 1.6 of EFV), IIA for Lacto-Cur-nano (59% ± 1.34) and IIIB for Lacto-EFV-nano (58.4% ± 1.79). This suggests that maximum amount of drugs has been entrapped in protein. It has also been observed that combination of EFV and Cur are synergistic in loading of one drug to the other.

### pH dependent release of drugs from NPs

**Figure 3:**
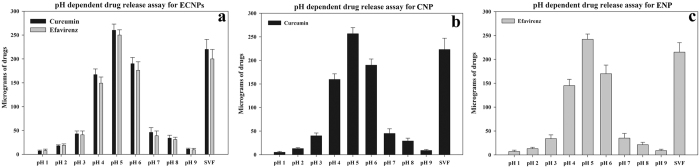
pH and simulated vaginal fluid dependent release profile of curcumin and efavirenz from ECNPs (**a**), curcumin from CNP (Lacto-nano-curcumin) (**b**) and efavirenz from ENP (Lacto-nano-efavirenz) (**c**). SVF represent simulated vaginal fluid. Each data points were repeated in triplicate (n = 3) and presented as Mean ± standard deviation (S.D).

ECNPs were incubated at different pH conditions to mimic the *in vivo* environment of rat vagina; 300 μg of drug loaded nanoparticles were incubated with different pH values (1–9) of PBS and simulated vaginal fluid (SVF). Results showed that ECNPs are more sensitive at pH 5 and 6 with maximum drug release observed at pH 5 (Fig. 3). All three types of nanoparticles either in single or combination form showed more than 80% of drug release at pH 5. At pH below 4 and above 6, only 10% of drug release was found. Thus suggests that nanoparticles slowly release drugs in the vaginal lavage in the pH range of 4 to 4.5. Furthermore, higher concentrations of Cur and EFV will be released at ≥pH 4.5, a condition where higher virus infectivity was detected in vaginal lavage^40^. In addition, bacterial vaginosis related-pH increase was seen which allows virus shedding^41^ and under these pH conditions ECNPs release higher concentrations of cur and EFV thus promoting higher viral neutralizing environment.

### Stability studies of nanoparticles-*in vitro*

The stability of ECNPs in PBS (phosphate buffer saline, pH7.4) suspension form was analyzed for at least 20 days at 4 °C and 25 °C. Data presented in Supplementary Table 1 shows that all the four parameters were found to be quite steady at both temperatures. An average negative charge of −25 mV and a PDI of 0.4 indicates the high stability and homogenous colloidal solution property of nanoparticles. The loading efficiency and size distribution of ECNPs were found to be reasonably constant.

### Anti-HIV activity of nanoparticles

Virus inhibition in the presence of nano forms either single or in combination remains sustained for a period up to 24 h of prior-exposure to cells while soluble forms show a slight decrease in activity upon this pre-exposure (Supplementary Fig. S1).

### Pharmacokinetic (PK) study result in vaginal lavage

**Figure 4:**
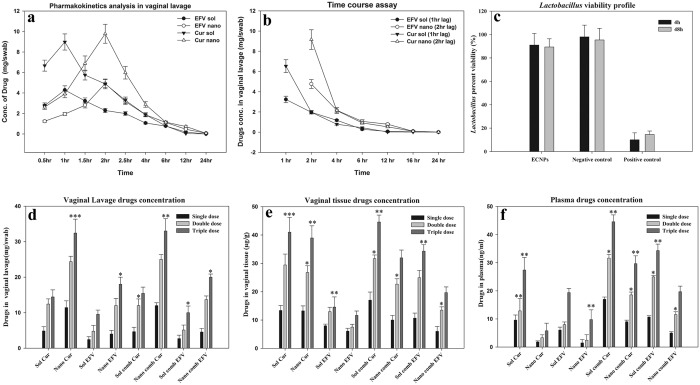
(**a**) Pharmacokinetics study: 20 mg of sol curcumin plus 10 mg of sol EFV and an equivalent amount of ECNPs were applied as single dose in vagina for indicated time points. (**b**) Time course experiment: same drug doses have been topically applied at a lag period of 1 h (for sol EFV + Cur) and 2 hr (for ECNPs). Lavages were collected after these time points and curcumin and efavirenz concentration were calculated separately. Abbreviation: EFV sol and Cur sol – concentration of EFV and Cur when delivered via Sol (EFV + Cur). EFV nano and Cur nano – concentration of EFV and Cur when delivered via ECNPs. (**c**) Viability percentage of *Lactobacillus cripatus* when treated with ECNP, at 4 h and 48 h, media without ECNPs and 1% triton X served as negative and positive control respectively. (**d–f**) Dose-dependent study of drugs in single form or combination form either in soluble EFV + Cur or ECNPs. d, e and f represents the concentration of drugs in vaginal lavage, vaginal tissue and plasma respectively. Sample data were recorded as Mean ± SD, n = 3 and value of significance expressed as ***P < 0.0005, **P < 0.005, *P < 0.05. Abbreviation: EFV-comb-sol & Cur-comb-sol: - efavirenz and curcumin concentration delivered as soluble combination. EFV-comb-nano & Cur-comb-nano: - efavirenz and curcumin concentration delivered via ECNPs. EFV-sol: Soluble EFV. Cur-sol: Soluble Cur. EFV-nano: EFV released form ENP. Cur-nano: - Cur release form CNP.

Curcumin and efavirenz levels were measured separately in vaginal lavage after administration of single dose of combination drugs either in soluble or nanoform. Drug levels were observed at nine different time points up to 24 h (Fig. 4a). We found enhanced PK profile (Table 2) when drugs were delivered via nanoparticles. The lavage PK shows more than 3-fold increase in AUMC both in the case of efavirenz as well as curcumin. Similarly there was more than 2-fold increase in T_max_ and t_1/2_ (Table 2). AUC values were also seen to be increased by 50% when nanoparticles were used as delivery system. This PK profile suggest the higher bioavailability of drugs when given through lactoferrin nanoparticles.

### Evaluation of drug release kinetics of ECNPs

To investigate the extent of availability of drugs over a period of long time, the time-dependent release study (study 2) was performed. The time-dependence was studied individually for the two drugs curcumin and efavirenz delivered in ECNPs; the results are shown in in Fig. 4b. The figure represents that the concentration of curcumin and efavirenz in vaginal lavage at various time point, after one (sol drugs combination) and two (ECNPs) hours of lag phase of drug administration. The results suggest high concentration in the initial stage for both the sol and nano form, which got decreased later exponentially. The nano formulations of drugs even after 2 hr of lag phase showed sustained and significant release of drug up to 12 hr whereas both the drugs in the sol form, showed fast reduction (at 4hr) in the concentration. We have indeed detected the presence efavirenz and curcumin up to 8–12 h, when drugs were given in nanoformulation but in case of the soluble form, the drugs were eliminated in 4–6 hrs.

### Viability assay profile of *Lactobacillus*

*Lactobacillus* is the most colonizing bacteria in the vagina, and is generally considered as a gatekeeper of the vaginal ecosystem. Any microbicide could be considered as safe until it is non-toxic to the growth of *Lactobacillus*. *Lactobacillus* naturally produces hydrogen peroxide which provide a natural barrier for HIV transmission. From Fig. 4c it is clear that there is no difference in the viability of bacteria between the media control and ECNPs, which confirms that ECNPs could be a safe microbicide.

### Dose dependent toxicity and bioavailability studies

#### Assessment of efavirenz and curcumin in vaginal lavage

The fixed doses mentioned in dose schedule (study 1) were administered intravaginally and the drugs estimated in lavage are plotted (Fig. 4d). Results suggest that the nano formulation of drugs either in single form or combination showed 1.8 (1^st^ dose) to 2.2 (3^rd^ dose) fold more availability of drugs at the topical site in a dose-dependent manner.

#### Efavirenz and curcumin concentration in vaginal tissue

Absorption of drugs in local cervical-vaginal tissue has been estimated and represented in Fig. 4e. Efavirenz and curcumin were found in very low concentrations in the range of micrograms, 1000-fold less than vaginal lavage. Cur NPs and EFV NPs didn’t show appreciable difference in exposure of drugs as compared to sol counterpart. But in the case of ECNPs, we found there was 1.2 (1^st^ dose) to 1.8 (3^rd^ dose) fold lower drug concentrations as compared to sol drugs in combination.

#### Systemic bioavailability of efavirenz and curcumin

Figure 4f represents the nature of accumulation of drugs in the systemic circulation. We found that the sol form of drugs either in single or combination showed 1.5 (1^st^ dose) to 1.7 (3^rd^ dose) fold more accumulation in plasma as compared to its nano form; leading to higher organ-associated toxicity in the case of sol formulation. In the comparison study between the ECNPs and its soluble combination form at the third dose, curcumin and efavirenz were found to be approx. 30 ng/ml & 20 ng/ml and 44 ng/ml & 35 ng/ml respectively, suggest that at higher doses also the NPs could me marked as safe.

#### Systemic and vaginal Proinflammatory cytokine response

**Figure 5:**
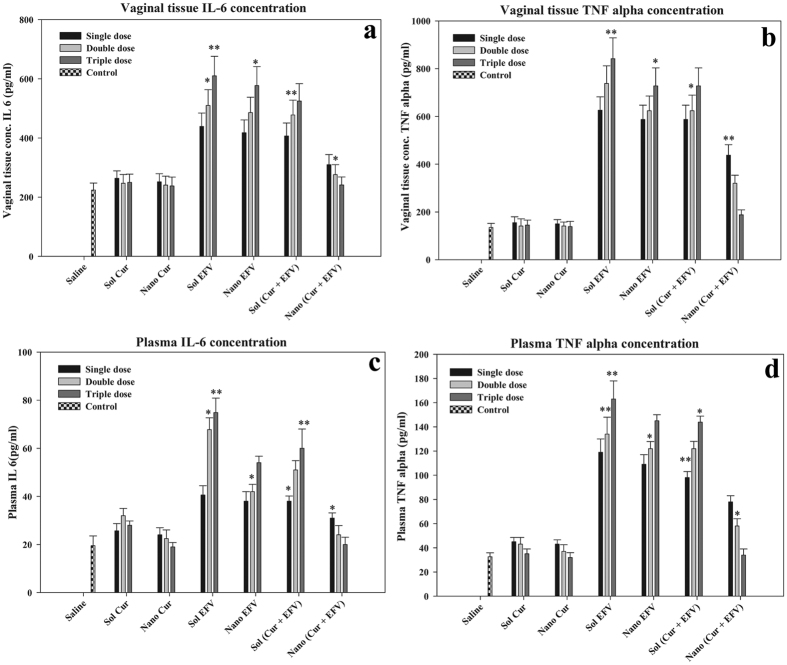
Dose dependent cytokine response. (**a**,**c**) Represent the IL-6 concentration in vaginal tissue and plasma respectively. (**b**,**d**) Represent the TNF-α concentration in vaginal tissue and plasma respectively. Sol Cur, Sol EFV, CNP, ENP, Sol (Cur + EFV) and ECNPs were applied topically in vagina and IL-6 and TNF-α were estimated. Value of significance ***P < 0.0005, **P < 0.005, *P < 0.05.

The proinflammatory cytokine markers; interlukine-6 (IL-6) and tumor necrosis factor alpha (TNF-α) levels were estimated in isolated plasma and vaginal tissue. Figure 5a,c represent the IL-6 level in vaginal tissue and plasma respectively. Sol EFV and Nano EFV showed at least three-fold increase in the level of IL-6. When EFV was encapsulated into lactoferrin together with curcumin, the IL-6 levels significantly decreased by 2.5 fold as compared to its sol formulation. At the third dose of ECNPs, we couldn’t detect any inflammation, in contrast to Sol (Cur + EFV) which leads to more than three-fold increase of IL-6, both in the vagina as well as in blood. Figure 5b,d represents the TNF-α level in vaginal tissue and plasma respectively. TNF-α is a major cytokine which stimulates the inflammatory response and leads to many autoimmune diseases and other clinical problems. In the case of ECNPs and Lacto-Cur-nano at the third dose, the TNF-α levels was found significantly decreased. These results suggest that nanoformulation provides an effective anti-inflammatory environment along with anti-HIV-1 drug EFV in vagina for protection from infection.

### Histopathological analysis

**Figure 6:**
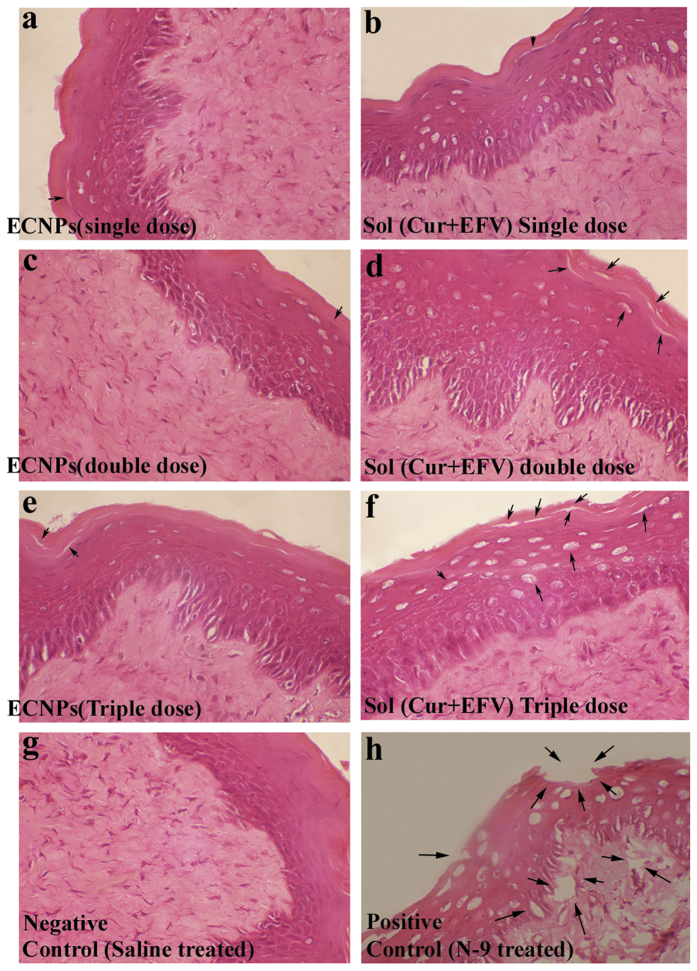
Histopathology vaginal tissue Hematoxylin and Eosin staining images. Left panel (**a**,**c**,**e**) represent the vaginal epithelia treated with ECNPs at first, second and third dose respectively. Right panel (**b**,**d**,**f**) represent the vaginal tissue integrity when applied topically with Sol (Cur+EFV) at first, second and third dose respectively. Damaged site or lesion were shown through a printing arrow. Negative (**g**) and positive (**h**) Controls were treated with saline and nonoxynol-9 (10 mg/kg) respectively.

Histopathological studies of cervicovaginal epithelia showed that ECNPs causes a reduced amount of toxicity in a dose dependent manner. The integrity of epithelia was found heavily damaged when the combination of sol drugs were used. In the case of ECNPs, the integrity of tissue was found to be same as that of control or very lesser extent. Tissue section pictures revealed that even at higher dose, ECNPs caused very less amount of tissue damage and at the same time soluble drug combination shows higher tissue damage. Negative (Fig. 6g) and positive controls (Fig. 6h) were treated with saline and nonoxynol-9 respectively. Left panel (Fig. 6a,c,e) represents the vaginal epithelia treated with ECNPs at first, second and third dose respectively. Right panel (Fig. 6b,d,f) represent the epithelia lining treated with Sol (Cur + EFV) at first, second and third dose respectively.

## Discussion

While several of the existing MPT have their own limitations^42^,^43^,^44^,^45^, currently the microbicide based MPT would be one of the finest products which could be adopted against HIV. Our study shows that, Protein nanoparticles formulation comprising of a combination of drugs would form one of the multipurpose prevention technologies (MPT) against HIV transmission. Curcumin and efavirenz loaded lactoferrin were developed using the sol-oil method, with a simple and fast approach without causing any chemical modifications to the drugs or vehicle protein^46^. In this method, a liquid-solid interface is formed from small molecules under macromolecular assemblies. Protein aggregation at this solid-liquid interface was prevented by sonication followed by exposure at low temperature. Particles could be easily precipitated and washed, and at this stage, particles can be stored over very long period even at room temperature. At room temperature this nanoparticle suspension appears as a collection of uniformly dispersed particles (Ex. Colloid). This methodology offers a possibility to produce various materials with novel, predefined properties in a very simple process and relatively at low cost. The main advantage of this method is to produce particles with very high purity and uniform nanostructure as assessed through FE-SEM and AFM analysis. In both types of nanoformulation, either blank or drug/s loaded, the DLS sizes were found to be greater than their SEM or AFM sizes (Fig. 1). The reason for this is quite rational because here the size was measured using light scattering method that could be affected by interaction of nanoparticle with the water shell and charge present on the surface that may lead to overall increase in size. ECNPs with a polydispersity index (PDI) of 0.435 are found to as homogeneous mixture. ECNPs was found to be more stable up to 20 days, as the parameters related to stability are found to be quite constant. This shows the versatile character of lactoferrin nanoparticles (Supplementary Table 1).

FT-IR data suggest the stability of efavirenz and curcumin in their nano form, as the majority of characteristics bands are still conserved (Fig. 2). In case on ECNPs all the major functional groups related to curcumin, efavirenz and lactoferrin were remains constant or little shift in the peaks may be observed. This shift is caused by the dipole moment present in the molecules, as the bond characteristics is electrostatic in nature. Results of this study showed that more than one drug can be encapsulated into the protein. Furthermore the study show a co-operativity in loading by synergistic action and molar equivalences. Since HIV-1 can acquires resistance to different combination of drugs and such a resistance in some cases is patient-dependent and this necessitates the design and development of formulation that include multiple drug regimens with multi-targeting capabilities. As curcumin and efavirenz exhibits very poor aqueous solubility, encapsulating these two drugs onto lactoferrin could solve the major issue of solubility limitation and the ECNPs designed, thus targets with its triple broad spectrum action comprising of curcumin, efavirenz and lactoferrin. Besides its anti-inflammatory properties, curcumin activity helps in inhibition of HIV-1 replication by binding to integrase and protease^47^ thus providing additional specificity to formulation. While efavirenz targets the Reverse transcriptase enzyme and the lactoferrin blocks virus entry^48^. In addition, lactoferrin acts as an immune modulator^38^ and exhibit broad spectrum anti-microbial activities^36^. Thus, this formulation would serve as a typical broad spectrum HIV microbicide. The main observation is the antiviral activity was enhanced for ECNPs when compared to their soluble and nanoforms individually (Supplementary Fig. S1). Here the maximum activity was obtained that is up to 98% even when the individual concentration of Curcumin, Efavirenz and Lactoferrin was reduced to half. This gives the advantage of using the drugs in combination with the nanoform there by reducing the dosage leading to the prevention of unwanted side effects and toxicities. The emergence of multi-drug resistance has led to an increase in failure of HIV treatment^49^. The vaginal lavage bioavailability studies showed an enhanced PK profile of drugs. The efavirenz and curcumin AUC (area under the curve) were found to increase by more than 50% when delivered via nanoparticles, thus pointing out that drug in nanoformulation gets exposed over long time to the vagina as compared to soluble formulation thus facilitating a giving long lasting protection against HIV. Similarly the other PK parameters such as T_max_ and t_1/2_ show a 2-fold increase, suggesting an enhanced stability of encapsulated drugs in nanoparticles (Table 2). Further, in the time course study ECNPs showed minimum burst effect suggesting that the drugs were released slowly and steadily Results of two hr. time lag indicated better performance (for nano drugs) as compared to one hour (for sol drugs) (Fig. 4b). ECNPs were found to release drugs up to 8–12 h while in soluble combination 70% of drug were eliminated from application site rapidly. The reason behind this could be that the nanoparticles are getting adsorbed on to the surface of vaginal epithelium and then releasing the drugs steadily into lavage^50^.

Lactoferrin is also found in its natural form in human vaginal secretion with premenstrual concentration of 3.8–11.4 μg/mg which after menopause increases up to 62.9–218 μg/mg^51^. Owing to such abundance in vaginal secretions, lactoferrin in ECNPs, may escape its recognition by system, thus is not the source of inflammatory response. Furthermore, curcumin down regulates inflammatory responses induced by EFV, which may be one of the reasons of low inflammatory response as compared to soluble combination in single dose as well as in multiple dose application (Fig. 5). Thus during ECNPs topical application, the major inflammation caused by EFV is not further enhanced and indeed is neutralized by the presence of lactoferrin together with curcumin. Hence, EFV will get a hassle free environment and it will exhibit maximum antiviral properties in lavage^52^. Lower concentrations of drugs were present in systemic circulation viz. nanograms of drugs concentrations, which was 10^−6^ fold lower than that observed in lavage and 10^−3^ fold lower than that in vaginal tissue and at these concentrations EFV may not show any significant toxicity. In contrast when soluble drugs are employed significant amount of drug was found to be present in the plasma as well as in tissue indicating the risk of using soluble EFV for vaginal application. Due to low toxicity to the *Lactobacillus* exhibited by ECNPs, there may not be any threat to vaginal microflora if ECNPs are used, which is a key marker for the safety of any microbicide (Fig. 4c). Furthermore, ECNPs delivered more quantity of drugs as compared to its soluble counterpart at all the dose levels. The important feature of microbicide based drug usage is to ensure sustained and controlled drug delivery in the vaginal environment without altering the vaginal epithelium, a critical requirement that could be achieved by ECNPs. Previous studies show that intravaginal application of microbicide causes the toxicity of local cervicovaginal epithelial tissue which damages it and finally leads to loss of its integrity^53^. Damaged tissue causes the more exposure to virus and leads to more viral invasion and infection.

In conclusion, lactoferrin loaded curcumin and EFV nanoparticles serves as control release formulation with low toxicity and higher bioavailability.

## Conclusion

In this study, curcumin and efavirenz were successfully encapsulated through sol-oil method and used as a controlled delivery system through vaginal route as a microbicide. The EE% and drugs release assay showed that vaginal pH is favorable for drug release. ECNPs showed less toxicity to *Lactobacillus cripatus* and vaginal epithelial tissue and release active drug entities in the vaginal lavage. In summary, lactoferrin loaded curcumin and EFV nanoparticles serves as a highly effective and efficient multi-protective and microbicide formulation.

## Materials and Methods

### Reagents

Curcumin and efavirenz were of 94% and 98% purity respectively. Lactoferrin was purchased from Symbiotics (Irvine, CA 92614 USA). Extra virgin olive oil was from Leonardo. All reagents and chemicals used were of analytical grade. IL-6 and TNF-α measurement kit were purchased from BD bioscience with cat no. 550319 and 558535 respectively

### Animals

In this study we have used healthy female albino Wistar rats. Experiments were carried out in accordance with the approved guidelines. All experimental protocol were approved by Institute Animal Ethics Committee, University of Hyderabad. Except rats used for tissue isolation, all other rats were reused after a wash period of 48 hours.

### Protein nanoparticle preparation

Lactoferrin nanoparticles (NPs) were prepared with the combination of curcumin and efavirenz through sol oil chemistry (patent filed)^26^. 40 mg of lactoferrin was dissolved in 500 μl ice cold PBS pH7.4. In a separate tube, 20 mg of curcumin was dissolved in 100 μl of DMSO and varying concentration (5, 10, 15, 20 mg) of efavirenz was dissolved in 100 μl of DMSO. Drug mixture was added to lactoferrin and incubated on ice for 1 hr. The sample was gently added to 25 ml of ice cold olive oil under continuous stirring. The mixture was sonicated on ice for 15 minutes using ultrasonic homogenizer with a pulse period of 30 sec and an amplitude of 5 μm at an interval of one min between sequential pulses. This mixture was frozen in liquid nitrogen for 15 min followed by 4 hr incubation on ice. The mixture so formed was centrifuged at 6000 g for 15 min; supernatant (containing olive oil) was discarded and the pellet was extensively washed with ice-cold diethyl ether to remove traces of oil. The pellet obtained was resuspended in PBS. The same protocol was followed for the preparation of individual nanoparticles of curcumin or efavirenz in different ratios mentioned in Table 1. Four different types of NPs were prepared (Table 1) for the present study; 1. Lactonano (without any drug), 2. Lacto-nano-EFV + Cur (ECNPs), 3. Lacto-cur-nano (CNP) and 4. Lacto-EFV-nano (ENP).

### Characterization of nanoparticles

Nanoparticles assembled were morphologically characterized using Field Emission Scanning electron microscope (FE-SEM) (Philips XL-30), Atomic Force Microscopy (AFM), Transmission electron microscopy (TEM). For SEM, particles were coated with gold. For TEM, samples were fixed on 200 mesh type-B copper grid coated with carbon (TED PELLA, INC.) and staining was done using 2% Uranyl acetate. Particles characterized through AFM (SPM400) were spin coated on a cover slip. Hydrodynamic radaii were measured using nanopartica nanoparticle analyzer, SZ-100 (Horiba Scientific).

### Fourier transform infrared spectroscopy (FTIR) of nanoparticles

FT-IR studies have been performed using ALPHA’s Platinum ATR single reflection diamond ATR module (Bruker Corporation). Briefly one to two milligrams of lyophilized samples were directly kept on the sample holder and scanned from 500 cm^−1^ to 4000 cm^−1^. The spectra were visualized using OPUS software. Base line correction has been done according to manufacturer instruction.

### Calculation of loading efficiency

Nanoparticles were incubated with 1 ml of 1X PBS (pH5) and kept under gentle rocking for 30 minutes at room temperature. 100 μl of 30% silver nitrate was added to resulting mixture to precipitate the protein. Then 1 ml of HPLC grade methanol was added to the mixture and this was centrifuged at 12000 rpm for 15 min and concentration of drugs in supernatant were estimated. Supernatant was analyzed in triplicate. Standard curve was developed using the different concentrations of curcumin and efavirenz through HPLC respectively. Loading efficiency was calculated as per equation (1).


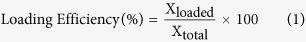



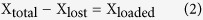


where X_loaded_ = amount of drug loaded, X_total_ = amount of total drug used and X_lost_ = amount of drug lost during preparation.

### *In vitro* experiments

#### Analysis of sensitivity of NPs under different conditions of pH and simulated fluid

Nanoparticle pellet were resuspended in 1 ml of 1X PBS (of different pH values in the range 1–9) and simulated vaginal fluid; These were then kept for incubation on rocker at room temperature for 4 h. Then 300 μl of 30% silver nitrate was added and drugs were extracted by adding 1 ml of methanol. These were centrifuged at 12000 rpm for 15 min and the supernatant was filtered using 0.2 micron syringe filter and analyzed by using HPLC. Every experiment was conducted in triplicate.

### *In vitro* stability profile of nanoparticles

The stability profile of nanoparticles was performed for ECNPs in suspension form and represented in Supplementary Table 1. The stability was measured in terms of the loading efficiency, size distribution, zeta (**ζ**) potential and polydispersity index (PDI). Suspension ECNPs were incubated for indicated time points such as 0, 1, 2, 4, 6, 8, 10, 12, 14, 16, 18 and 20 days at two different temperature (4 °C and 25 °C). Loading efficiency was calculated according to equation (1). Size distribution, PDI and zeta potential were measured using dynamic light scattering methods.

### Anti-HIV assay

Anti-HIV-1 activity of Lacto-Cur-nano, Lacto-EFV-nano and ECNPs along with their soluble counterparts was analyzed using HIV-1 NL4-3 virus clone. The concentrations of Curcumin, Efavirenz and Lactoferrin were taken as 10 μM, 2 nM and 1 μM respectively. But the concentrations of all the drugs and Lactoferrin were reduced to half in the preparation of ECNPs The experiment is conducted by adding drug to Sup T-1 cells and incubating for 1, 3, 4 and 8 hours respectively. After incubation, cells were washed and challenged with HIV-1_NL4-3_ and incubated for 5 days and virus replicated was estimated at day 5 using HIV-1 p24 antigen capture assay (ABL). Based on the virus replicated in the control in absence of drug, drug efficacy was measured in-terms of percent inhibition of HIV-1 replication.

### *In vivo* experiments

#### Animal experimental design

Animals (0.160–0.240 kg, 6months) were housed for seven days prior to experiment at the animal housing facility, University of Hyderabad for 12 h in a light/dark condition. Animals were euthanized using sodium pentobarbital (50 mg/kg, IP) and final sacrifice was done by cervical dislocation. Blood was collected through heart puncture during terminal anesthesia. All the animal experiments were done in triplicate. All the experiments were performed on healthy female rats. 54 rats were used for pharmacokinetics (PK) study (study1) and these were reused for study 3 after a wash period of 48 h. 6 rats were used for time course bioavailability studies (study 2), and among these, 3 rats were randomly selected and reused for study 3. Study3 (dose dependent toxicity and bioavailability studies) were performed on pooled rats from study 1 and 2. An outline of all the animal experiment is provided in Supplementary Fig. S2.

### Dosage schedule

For studies 1 to 3, drugs used were in the combination of 20 mg of curcumin (sol curcumin) plus 10 mg of efavirenz (sol efavirenz) and an equivalent amount of combination nanoformulation per rat. For single drug, curcumin (20 mg) or EFV (10 mg) was used either in sol or nano form. The best formulation ratio such as II, IIA and IIIB as given in Table 1 were used for this study. Nano formulation of drugs used in this experiment was equivalent to its sol form. All the drugs were topically applied in vagina. Saline treated rats were considered as control.

#### Pharmacokinetic assessment in vaginal lavage

For study 1, Single dose of curcumin plus efavirenz either in sol combination or nano-combination as mentioned in the dosage schedule were topically applied in vagina to different groups of animals. After completion of various time points such as 0.5, 1, 1.5, 2, 2.5, 4, 6, 12 and 24 h, vaginal lavage was collected using sterile cotton swabs. Further, the swabs were mixed with methanol and the drug was extracted. Drugs were quantified using HPLC at the above mentioned time points; pharmacokinetics parameters were calculated using Kinetica v.5 software.

#### Time course experiment *in vivo*

Sustained release of drugs in vaginal lavage: For study 2, all the animals were administered with single dose of Sol (EFV+Cur) and nano (EFV+Cur) or ECNPs. On the basis of T_max_ obtained from PK study, the time course study was performed with a lag period of 1 hr (for sol drugs) and 2 hr (nano drugs). The vaginal lavages were manually collected at fixed time points viz. 30 min, 1 h, 2 h, 4 h, 8 h, 12 h, 16 h and 24 h with sterile cotton swabs. Drugs present in swabs were extracted in methanol and estimated for efavirenz and curcumin separately using HPLC.

#### *Lactobacillus* viability assay

To evaluate the effect of ECNPs on the growth of *Lactobacillus crispatus.* Lactobacillus viability test was performed according to standard protocol^54^. The Bacterial density of 0.06 (OD at 670 nm) or 108 CFU/ml (100 μl) was seeded into a sterile 96 well plate and incubated with 100 μl of 1 mg/ml of ECNPs at 37 °C. Media without ECNPs was considered as negative control and triton X (1%) as a positive control. After 4 and 48 h of incubation, 20 μl of MTS reagent was added and absorbance was measured at 490 nm. The percentage viability was calculated using the formula; viability percentage = (absorbance of Test sample/absorbance of Control sample)/100. Where absorbance of Test and Control samples is represented by the quantity of formazan reduced by viable cells.

#### Dose dependent toxicity and bioavailability studies

For study 3, a dose dependent study was performed to examine the novelty of nanoparticles as a microbicide. The study was carried out to compare the effect of sol and nano form of drug either in single or combination form. In total six type of formulations, such as Sol Cur, Nano Cur, Sol EFV, Nano EFV, Sol (Cur+EFV) and ECNPs were used and the study was carried out up to three doses. Doses were applied according to the schedule described below at an interval of 2 h between each dose. After the completion of each dose, rats were sacrificed under proper anesthetic condition. Vaginal lavage, blood, and vaginal tissue were collected. Blood was collected through cardiac puncture. Plasma was isolated from blood and used for the estimation of drugs. Vaginal tissues were collected and used for the estimation of inflammatory markers. Cytokines (IL-6 and TNF-alpha) levels were estimated in plasma and in the vaginal tissue homogenate. A small part of vaginal tissue were saved in 10% neutral buffered formalin and further processed for histopathology through Hematoxylin and Eosin tissue staining. Major part of vaginal tissues were homogenized in PBS using homogenizer. After final processing of vaginal tissues, blood plasma, and vaginal lavage; concentrations of curcumin and efavirenz accumulation were determined using HPLC.

#### Drug extraction from vaginal tissue

The Vaginal tissue collected was homogenized in PBS using rotor stator homogenizer for 15 minutes. 300 μl of 30% silver nitrate per ml was added to the tissue extract to precipitate the protein. Drugs were extracted using 3 ml of HPLC grade methanol. Then curcumin and efavirenz were estimated through HPLC at 425 nm and 247 nm respectively.

### Mobile phase chromatographic condition

A reverse phase C18 column (25 cm × 4.60 mm, particle size 5 μm) (Purospher® STAR RP-18 endcapped (5 μm) Hibar® RT 250-4.6, column No. 148837, Merck Millipore) was used for HPLC analysis. The mobile phase for efavirenz and curcumin were as follows. For efavirenz, mobile phase consists of 25% of 0.1% formic acid (Milli-Q water, pH 3.2) and 75% of acetonitrile, and the flow rate was set to 0.3 mL/min at ambient temperature^55^. For curcumin the mobile phase is composed of acetonitrile: 5% acetic acid in the ratio of (75:25, v/v)^56^. All solvents were filtered using 0.4 μm nylon syringe filter and degassed prior to use. 10 μl of sample were analyzed for detection of EFV and curcumin.

### Histopathology study

The Histopathology study was done only for combination form of drugs either in soluble form or nanoformulation (ECNPs). Doses of soluble drugs in combination and ECNPs were given as described in dose schedule (n=3). Negative and positive controls animals were treated with saline and 10 mg/kg nonoxynol-9 (N-9) respectively. Multiple doses were repeated up to three doses at time gap of 2 h. After the completion of measurements at the time points, animals were sacrificed under standard protocol. Vaginal tissue was fixed in 10% Neutral Buffered Formalin, followed by sectioning using cryomicrotome and Hematoxylin& Eosin staining. All pictures included in the results section were taken at 100× zoom using Olympus BX51P polarizing microscope.

### Statistical analysis

All studies were performed in triplicate. Data were presented as mean and standard deviation. The significance difference were calculated using one way ANOVA. The level of significance was used as ***P < 0.0005, **P < 0.005, *P < 0.05.

## Additional Information

**How to cite this article**: Lakshmi, Y. S. *et al.* Triple combination MPT vaginal microbicide using curcumin and efavirenz loaded lactoferrin nanoparticles. *Sci. Rep.* **6**, 25479; doi: 10.1038/srep25479 (2016).

## Supplementary Material

Supplementary Information

## Figures and Tables

**Table 1:** Loading efficiency (LE) of drug(s) loaded lactoferrin nanoparticle.

Formulations	Lactoferrin concentration (mg)	Curcumin concentration (mg)	Efavirenz concentration (mg)	Loading Efficiency of curcumin	Loading Efficiency of efavirenz
LE of ECNPs					
I	40	20	5	47% ± 2.5	49% ± 2.4
II	40	20	10	63% ± 1.9	61.5% ± 1.6
III	40	20	15	57.4% ± 3.2	51.7% ± 2.7
IV	40	20	20	48% ± 2.8	53% ± 3.7
LE of Lacto-Cur-nano					
IA	40	5	0	38% ± 1.45	NA
IIA	40	10	0	59% ± 1.34	NA
IIIA	40	15	0	52% ± 2.2	NA
IVA	40	20	0	49.5% ± 2.5	NA
LE of Lacto-EFV-nano					
IB	40	0	5	NA	41% ± 1.73
IIB	40	0	10	NA	47.6% ± 2.8
IIIB	40	0	15	NA	58.4% ± 1.79
IVB	40	0	20	NA	57.83% ± 2

NA: Not applicable.

**Table 2:** Pharmacokinetic profile in vaginal lavage.

Parameters	Units	EFV*		Curcumin#	
		ECNPs	Sol (EFV + Cur)	ECNPs	Sol (EFV + Cur)
**AUC**	(h)*(mg/ml)	20.4164	13.7581	32.4251	21.9066
**AUMC**	(h)^2*(mg/ml)	132.482	52.448	172.922	56.496
**C**_**max**_	mg/mL	4.9126	4.2897	9.7794	8.9723
**T**_**max**_	hr	2	1	2	1
**t**_**1/2**_	hr	4.63222	2.14916	3.97347	1.8513

Pharmacokinetic parameters.

AUC: The integral of the concentration-time curve (after a single dose or in steady state).

AUMC: Partial area under the moment curve between t start and t end.

C_max_: The peak plasma concentration of a drug after oral administration.

T_max_: Time to reach C_max_.

t_1/2_: The time required for the concentration of the drug to reach half of its original value.

^*^EFV credentials measured individually when delivered via ECNPs and Sol (EFV+Cur) respectively.

^#^Cur credentials measured individually when delivered via ECNPs and Sol (EFV+Cur) respectively.
