# Supplementary material for: Triple combination MPT vaginal microbicide using curcumin and efavirenz loaded lactoferrin nanoparticles
Source: Sci Rep. 2016 May 6;6:25479. doi: 10.1038/srep25479 (PMC4858693; doi:10.1038/srep25479)

Triple combination MPT vaginal microbicide using curcumin and efavirenz loaded lactoferrin nanoparticles

Yeruva Samrajya Lakshmi[#,a], Prashant Kumar[#,a], Golla Kishore[a], C Bhaskar[a] and Anand K Kondapi[a]*

[#]Authors have contributed equally.

[a]Department of Biotechnology and Bioinformatics, School of Life Sciences, University of Hyderabad, Hyderabad-500046, India.

Anand K Kondapi- akondapi@gmail.com

*Corresponding author :

Anand K. Kondapi, Ph.D.

Professor

Department of Biotechnology and Bioinformatics

University of Hyderabad

Hyderabad 500046 India

Ph: [O] 91-40-23134571 ® 91-40-23000654

Cell: 91-9246212654

Fax: 91-40-23010145

Supplementary information

Supplementary figure

Fig. S1. Anti-HIV assay of nanoparticles. Sup-T1 cells were incubated with indicated condition for 1, 3, 8 and 24 hours. Then, cells were washed and challenged with HIV-1_{NL4-3}. Amount of virus was estimated on day 5 by p24 antigen capture assay. Percent inhibition of virus replication was estimated based control in absence of drug. Each data point is an average of three independent experiments.

Fig. S2. Animal study design: Female Wistar rats were randomly divided in to three different type of study (1-3).

Supplementary Table

Supplementary Table 1. Stability profile of ECNPs.

	Size[a] (nm)		ζ potential[b] (mV)		PDI[c]		Encapsulation Efficiency %			
**Days**	**4 °C**	**25 °C**	**4 °C**	**25 °C**	**4 °C**	**25 °C**	**4 °C**		**25 °C**	
							**Cur**	**EFV**	**Cur**	**EFV**
**0**	98±5.6	98±5.6	-19±3.2	-19±3.2	0.435	0.435	63%±1.9	61.5%±1.6	63%±1.9	61.5%±1.6
**1**	105±6.4	108±5.7	-21±4.1	-22±1.9	0.351	0.361	62%±2.1	60%±5.9	62.81%±2.4	61.27%±3.1
**2**	110±7.3	114±6.4	-24±3.8	-22±2.3	0.473	0.483	64%±3.2	62%±4.8	62.3%±3.30	60.3%±2.9
**4**	105±8.5	118±6.9	-20±4.6	-21±1.8	0.483	0.495	63%±2.3	62%±8.4	61.0%±2.70	60.75%±2.1
**6**	116±9.5	121±5.1	-21±2.3	-25±2.7	0.535	0.497	61%±5.4	60%±4.6	61.87%±3.1	60.61%±1.8
**8**	109±5.7	109±6.7	-17±1.9	-28±2.3	0.364	0.457	64%±4.9	62%±6.8	62.6%±2.55	60.83%±3.2
**10**	121±9.6	110±6.1	-24±2.8	-25±2.9	0.472	0.532	59%±7.4	60%±4.8	62.31%±1.9	59.7%±3.6
**12**	110±7.5	103±7.4	-25±1.7	-19±1.9	0.385	0.452	61%±5.1	60%±5.3	61.3%±2.25	59.57%±1.9
**14**	117±8.7	119±5.8	-27±3.1	-23±2.8	0.518	0.583	62%±4.9	60%±5.4	60.7%±2.56	59.85%±2.1
**16**	112±4.8	115±4.0	-30±2.8	-27±1.3	0.485	0.517	63%±3.9	62%±4.7	60.33%±1.7	59.18%±3.5
**18**	119±7.3	123±9.1	-26±1.5	-24±2.8	0.392	0.428	64%±3.2	61%±5.0	60.65%±3.6	58.97%±2.7
**20**	120±5.9	116±6.1	-19±2.8	-22±1.9	0.373	0.486	65%±6.1	63%±5.1	60.5%±2.25	58.77%±1.8

[a] Hydrodynamic radius measured by Dynamic light scattering.

[b] Zeta potential measured by zeta sizer.

[c] poly-dispercity index.

All samples were measured in triplicate. Data were presented as mean ± standard deviation.

Supplementary Table 2. FT-IR study profile.

	Featured band	Soluble formulation (wave number in cm^{-1})	Nano formulation (wave number in cm^{-1})
**Lactoferrin**	Amide I	1632.79	1641.52
	Amide II	1536.74	1538.32
**Efavirenz**	C-O	1073.45	1073.47
	CH2 stretch	1195.06	1195.43
	C=O	1744.04	1744.95
	C≡C	2248.72	2249.26
**Curcumin**	C-O-C	1024.62	1025.13
	enol C=O	1271.17	1273.00
	Aromatic ring	1600.74	1601.62
	C=O plus C=C	1626.20	1626.50
	–OH	3507.82	3501.99
**Efavirenz plus Curcumin**	C-O (EFV)	1073.61	1073.53
	CH2 stretch (EFV)	1200.88	1201.81
	C=O (EFV)	1745.90	1745.64
	C≡C (EFV)	2249.55	2249.58
	C-O-C (Cur)	1025.87	1026.75
	enol C=O (Cur)	1262.41	1274.47
	Aromatic ring (Cur)	1600.57	1601.46
	C=O plus C=C (Cur)	1626.41	1627.20
	–OH (Cur)	3508.15	3508.04

Supplementary Figure S1.

Anti-HIV assay of nanoparticles.

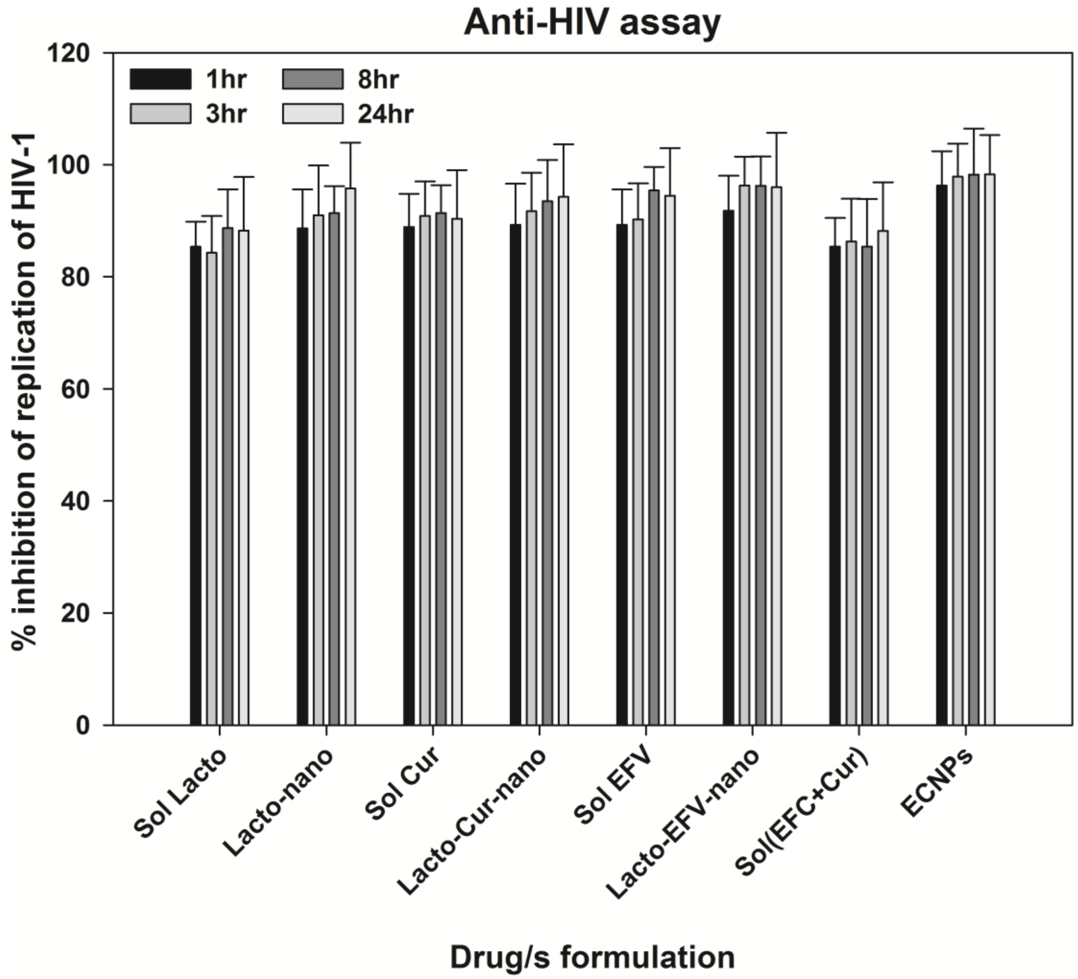

Supplementary Figure S2.

Animal study design:

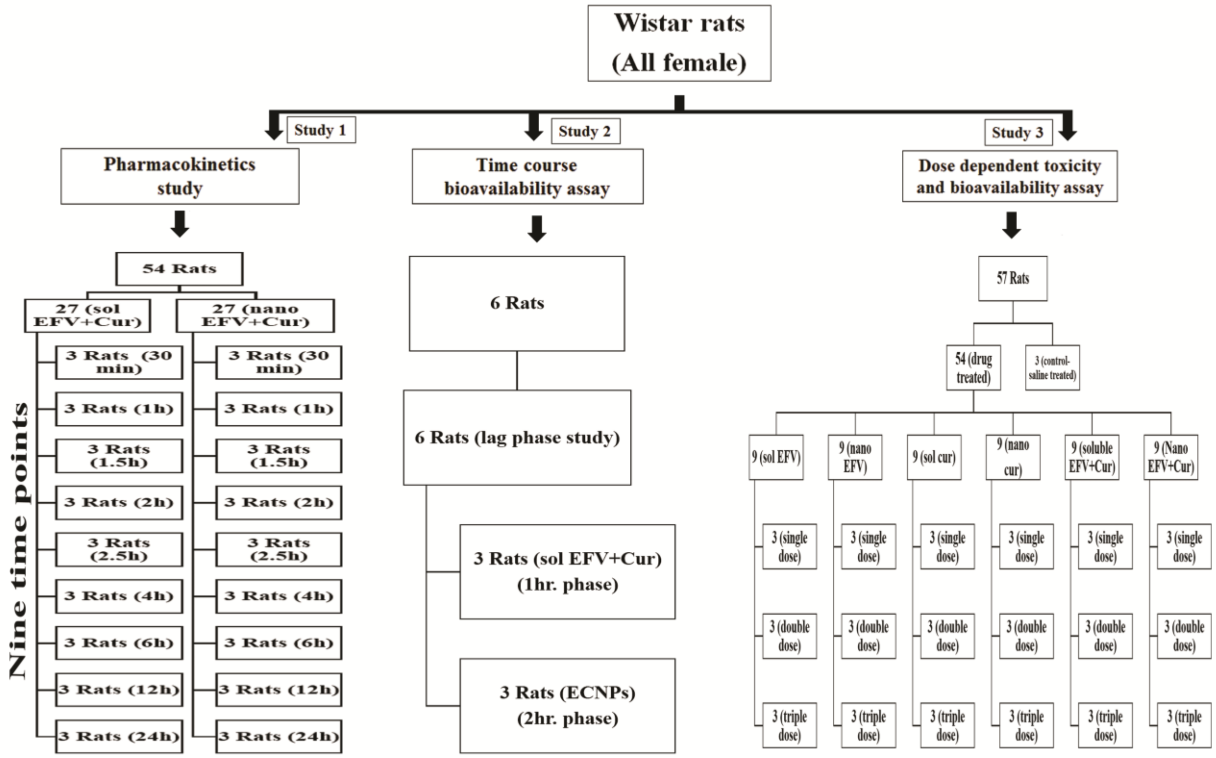

Supplement: Supplementary Information [file srep25479-s1.pdf]
